# Microheater with copper nanofiber network via electrospinning and electroless deposition

**DOI:** 10.1038/s41598-023-49741-7

**Published:** 2023-12-14

**Authors:** Na Kyoung Kim, Kanghyun Kim, Hansol Jang, Taechang An, Hyun-Joon Shin, Geon Hwee Kim

**Affiliations:** 1 Department of Mechanical Engineering, Chungbuk National University (CBNU), 1, Chungdae-ro, Seowon-gu, Cheongju-si, Chungcheongbuk-do 28644 Republic of Korea; 2 Department of Mechanical Engineering, Pohang University of Science and Technology (POSTECH), 77, Cheongam-ro, Nam-gu, Pohang-si, Gyeongsangbuk-do 37673 Republic of Korea; 3 Department of Physics, Chungbuk National University (CBNU), 1, Chungdae-ro, Seowon-gu, Cheongju-si, Chungcheongbuk-do 28644 Republic of Korea; 4 Department of Mechanical Robotics Engineering, Andong National University (ANU), 1375, Gyeong-Dong-ro, Andong-si, Gyeongsangbuk-do 36729 Republic of Korea

**Keywords:** Electrochemistry, Mechanical engineering, Electrical and electronic engineering, Electronic devices, Sensors, Nanowires

## Abstract

In this report, we present the development of a copper nanofiber network-based microheater, designed for applications in electron microscopes, gas sensing, and cell culture platforms. The seed layer, essential for electroless deposition, was fabricated through the electrospinning of a palladium-contained polyvinylpyrrolidone solution followed by a heat treatment. This process minimized the contact resistance between nanofibers. We successfully fabricated a microheater with evenly distributed temperature by controlling the electrospinning time, heat treatment conditions, and electroless deposition time. We assessed the electrical and thermal characteristics of the microheater by examining the nanofiber density, sheet resistance, and transmittance. The microheater’s performance was evaluated by applying current, and we verified its capacity to heat up to a maximum of 350 °C. We further observed the microheater’s temperature distribution at varying current levels through an infrared camera. The entire manufacturing procedure takes place under normal pressure, eliminating the need for masking or etching processes. This renders the method easily adaptable to the mass production of microdevices. The method is expected to be applicable to various materials and sizes and is cost-effective compared to commercially produced microheaters developed through microelectromechanical system processes, which demand complex facilities and high cost.

## Introduction

Microheaters have found utility in a variety of applications such as gas sensors, where they elevate the temperature to facilitate the reaction between the gas and the sensing material^1–3^. They are also used in cell culture platforms^4^, microfluidic chips^5^, wearable devices^6^, and infrared sources^7^. Miniaturizable heating mechanisms typically include ultrasonic heating^8^, radiative heating^9^, the Peltier effect^10^, and the Joule heating principle^11^. Of these, Joule heating, which involves a current flowing through a resistive wire, is the simplest.

Micro-Joule heaters necessitate high electrical resistance for efficient heating, making the selection of a suitable material crucial^9^. Frequently used metal materials include platinum^12^, titanium^13^, tungsten^14^, gold^15^, and copper^16^. Microheater substrates are primarily ceramic-based materials. For efficiency, the substrate in contact with the ground should possess low thermal conductivity, while the intermediary substrate sandwiched between the heating layer and the heat transfer material should exhibit high thermal conductivity^17^.

Microheaters employing the Joule heating method typically use processes such as physical vapor deposition (PVD)^18^, chemical vapor deposition (CVD)^19^, and electrochemical vapor deposition (EVD)^20^ to pattern elongated electrodes onto the substrate. PVD processes, including sputtering^21^ and electron beam evaporation^22^, are integral to semiconductor fabrication. These processes form a thin metal film on a substrate via the bombardment of ionized gas atoms on the targeted deposition material. They offer the advantage of depositing most metals and alloys in a thin layer^23^. Meanwhile, CVD involves forming a thin film on the substrate through a chemical reaction that produces the deposition material in gaseous form. Given its high adhesion to the target substrate and its applicability to most surfaces, it is a highly versatile technology^24^. EVD is a metallization method that leverages the electrolysis phenomenon, where anions move to the anode and cations to the cathode. It has recently been demonstrated to be applicable to insulators^25^. However, both PVD and CVD processes are typically conducted under vacuum^26^ and to fabricate a heater, a microelectromechanical system (MEMS) process is required. While EVD requires an external power source, it suffers from the limitation of poor uniformity^27^.

In certain applications, microheaters are required to be transparent. For instance, they are used in scanning transmission X-ray microscopes to heat particles loaded on a substrate to analyze their properties as a function of the annealing temperature^28^. Devices integrating a gas sensor, an LED, and a microheater have also been fabricated^29^.

Transparent electrodes, boasting high transmittance and electrical conductivity, find application in displays^30^ and solar cells^31^. The most widely used material for transparent electrodes is indium tin oxide (ITO). However, the increasing prominence of wearable and flexible devices has driven up the cost of ITO^30^, and its high brittleness^32^ is a concern. As a result, transparent electrodes can now be fabricated using carbon-based materials such as carbon nanotubes^33^ and graphene^32^, conducting polymers^34^, composite materials^35^, and metals such as gold^36^, silver^37^, and copper^38^ in nanowire form.

Nanowires, including those made of gold^39^, silver^40^, and copper^41^, can undergo electroless plating through oxidation–reduction reactions on the surface without any external current. The thickness and particle shape of the metal layer deposited using this method depend on factors such as pH^42^, temperature^43^, and deposition time^44^. The catalyst seed necessary for electroless deposition can be applied using electrospinning, a process that uses high voltage to produce micro- to nano-scale-diameter fibers from a polymer solution, driven by the repulsive force between electrical charges. The shape of the electrospun fibers can be controlled by manipulating parameters such as the viscosity of the electrospinning solution, spinning time, distance between the solution discharge and collector, and solution flow rate^45^. After seed layer application, fiber electrodes with diameters ranging from several nanometers to micrometers can be efficiently formed through electroless deposition^46,47^.

For in-situ annealing experiments in STXM analysis of metallic materials such as Co, Ni, the micro heating devices with high heat resistance and mechanical strength are generally used^48^. However, these microheaters are usually fabricated based on MEMS-based processes, but this process is expensive and need precise control of aligning the conducting pattern, which in turn increases the cost of the fabricated devices. In this study, we fabricated a miniaturized, low-cost, transparent microheater by sequentially executing electrospinning and electroless deposition on thin silicon nitride, which is typically used in X-ray evaluation experiments.

As a substrate of microheater, we used silicon nitride membrane, which has high heat resistance, light transmittance, durability so that generally used in various in-situ annealing experiments^49^. Other polymer-based film substrate might be degrading or damaged when used as a micro heater, heated up to the melting point of the polymer. Polyvinylpyrrolidone (PVP) fibers containing palladium were electrospun, and then the PVP was removed via pyrolysis to form a seed layer. Subsequently, palladium was electrolessly plated with copper—a material gaining attention for its use in microheaters and transparent electrodes—to achieve a highly transmissive microheater. Each fabrication method can be quantitatively controlled by adjusting the process time. After applying a DC current to the fabricated microheaters, we confirmed a successful temperature increase using an infrared camera. This fabrication method, which does not require a separate MEMS process, is anticipated to be adaptable for various materials and sizes.

## Experimental section

### Materials

A 5-μm-thick silicon nitride membrane (Si_3_N_4_; frame size: 5 mm × 5 mm, membrane size: 0.5 mm × 0.5 mm) was procured from Silson Ltd. (United Kingdom), while PVP (AR grade; molecular weight, 1,300,000) powder was sourced from Alfa Aesar (USA). Copper(II) sulfate pentahydrate (CuSO_4_·5H_2_O; special grade, 99.5%), *N*,*N*-dimethylformamide (DMF; special grade, 99.5%), sodium hydroxide (NaOH; special grade, 98.0%), ammonium tetrachloropalladate(II) ((NH_4_)_2_PdCl_4_; 99.998% metal basis), and potassium sodium tartrate tetrahydrate (KNaC_4_H_4_O_6_·4H_2_O) were procured from Sigma-Aldrich (USA). Formaldehyde solution (HCHO; 36.0–38.0%) was acquired from Wako Pure Chemical Industries, Ltd. (Japan). All reagents were used as received, without any purification.

### Characterization

The morphology of the nanofibers at different process stages was observed using an optical microscope (ECLIPSE LV150N; Nikon, Japan) and a field emission scanning electron microscope (FE-SEM; Ultra Plus; ZEISS, Germany). The properties of the nanofibers were analyzed with an energy dispersive X-ray spectrometer (FlatQUAD; Bruker, USA). The linewidth and area fraction of the fabricated nanofibers were measured using the ImageJ software (NIH, USA). The transmittance of the nanofiber structures, as a function of copper deposition time, was measured in the spectral mode of an ultraviolet–visible spectrophotometer (OPTIZEN POP-V; JASCO, Japan), using a bare silicon nitride membrane as the reference. The sheet resistance of the heat-treated/metallized nanofiber structure was measured using a four-point probe method (CMT-SR2000 N; AIT Co., Korea). To verify the performance of the fabricated microheater, a current was applied from a current source (2231A-30-3 Triple Channel DC Power Supply; Keithley, USA), and the resulting heating temperature was determined. The temperature distribution was observed using an infrared camera (FLIR A655sc 25°; Teledyne FLIR, USA) and the FLIR ResearchIR Max software (Teledyne FLIR, USA).

### Palladium seed layer deposition by electrospinning

**Figure 1:**
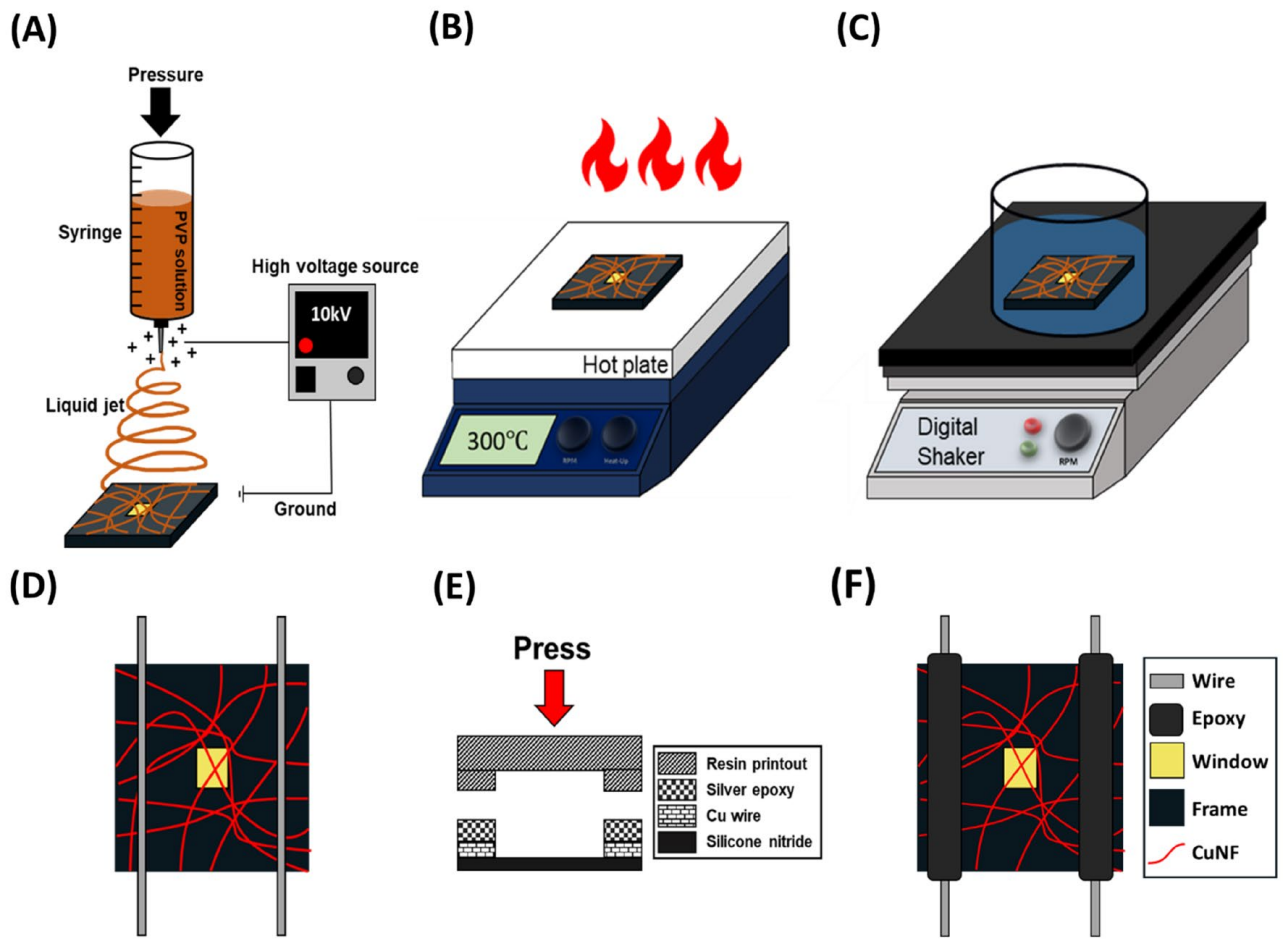
Schematic of the microheater fabrication process. (**A**) Electrospinning of PVP-palladium nanofiber onto a silicon nitride membrane. (**B**) Creation of a palladium-embedded seed layer through heat treatment. (**C**) Copper electroless deposition on the palladium-embedded seed layer using a chemical reaction. (**D**) Placement of a conducting wire on the copper-deposited nanofibers. (**E**) Application of pressure via a resin stone to firmly bond the conducting wire to the copper-deposited nanofibers. (**F**) Final, fabricated microheater.

An electrospinning solution was prepared by adding 0.3 g of ammonium tetrachloropalladate(II) and 3 g of PVP to 10 mL of DMF. This mixture was stirred at 300 rpm for 24 h using a vortex mixer. The silicon nitride membrane was used as a substrate and attached to the collector. Electrospinning was performed by applying 10 kV for 30 s, while maintaining a 10-cm distance between the syringe tip and the collector. During this process, relative humidity was kept between 40–50% and the temperature was controlled at 25–30 °C (Fig. 1A). Subsequently, the electrospun fibers were heat treated at 300 °C for 30 min to consolidate the intersection junctions and degrade the PVP gradually, forming a palladium seed layer (Fig. 1B). During this process, the nanofibers adhere well to the silicon nitride membrane so that the adhesive strength between consequently growing copper wire and the substrate^50^.

### Copper nanofiber formation by electroless deposition

Following seed layer deposition, a copper conductive layer was grown on the palladium-embedded seed layer. The copper electroless deposition solution was prepared by dissolving formaldehyde, sodium hydroxide, potassium sodium tartrate tetrahydrate, and copper(II) sulfate pentahydrate in deionized water at concentrations of 0.1 mL/mL, 40 mg/mL, 140 mg/mL, and 30 mg/mL, respectively. In this mixture, formaldehyde served as a reducing agent to supply electrons, sodium hydroxide was used to adjust the pH, potassium sodium tartrate tetrahydrate functioned as a complexing agent, and copper(II) sulfate pentahydrate supplied copper metal ions. The chemical equation for copper electroless deposition is as follows:

$${{\text{Cu}}}^{2+}+2{\text{HCHO}}+4{{\text{OH}}}^{-} \to {\text{Cu}}+2{{\text{HCOO}}}^{-}+2{{\text{H}}}_{2}{\text{O}}+2{{\text{H}}}_{{\text{ads}}}$$

The copper electroless deposition process underwent the Cannizzaro reaction for 2 min on a digital shaker at 50 rpm, at room temperature (Fig. 1C). A conductive wire was gently placed on a silicon nitride membrane and an electrical connection was established with a macroelectrode and silver paste (Fig. 1D–F).

## Results and discussion

### Analysis of morphological, electrical, and optical properties of the microheater

**Figure 2:**
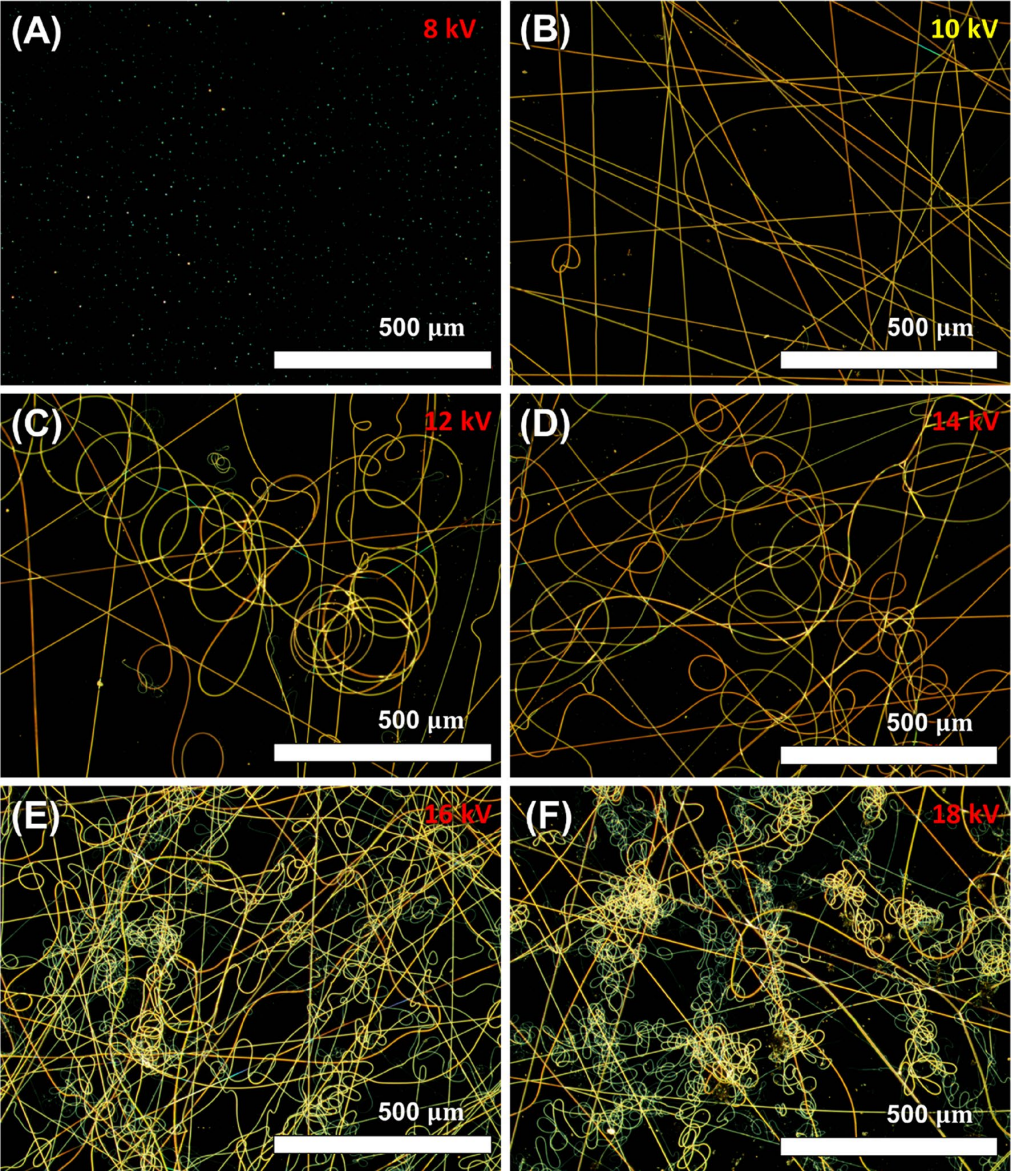
Comparison of fiber morphology against the applied voltage. (**A**) 8 kV, (**B**) 10 kV, (**C**) 12 kV, (**D**) 14 kV, (**E**) 16 kV, (**F**) 18 kV.

Figure 2 presents the comparison of fiber morphology against the different voltage applied. Because voltage directly affects to the amount of charge applied to the electrospinning solution, it strongly influencing the geometry of electrospun fibers. Figure 2A–F are results of the fabricated fibers by only increasing voltage applied to the PVP-Pd solution from 8 to 18 kV in 2 kV increments under same conditions, respectively. Under 10 kV of voltage was applied, the Taylor cone of the electrospinning solution didn’t form and the electrospinning process had not happened. But, when the 10 kV of the voltage was applied, the Taylor cone started to be formed so that the electrospinning process started, and the straight fiber network was fabricated. However, as the voltage was increased from 12 to 18 kV, the frequency and amounts of coil-shaped fibers rapidly increased due to the jet splitting. Increasing the voltage accelerates the polymer jet, which results in a larger volume of solution being ejected. Also, when the voltage higher than the critical voltage of the electrospinning process, the jet becomes highly concentrated around the tip of the needle instead of the Taylor cone^51^. The higher voltage causes the droplet of the solution recede into the needle tip. Also, this concentrated electric field results in ejection of multiple jet and leads to jet splitting. As a result, the multiple jet makes the collected fiber looks coils^52^. These coil-looked fibers results in an increase of overlapped part between fiber network, which lowers the electrical conductivity, making it an unsuitable structure for used as a microheater.

**Figure 3:**
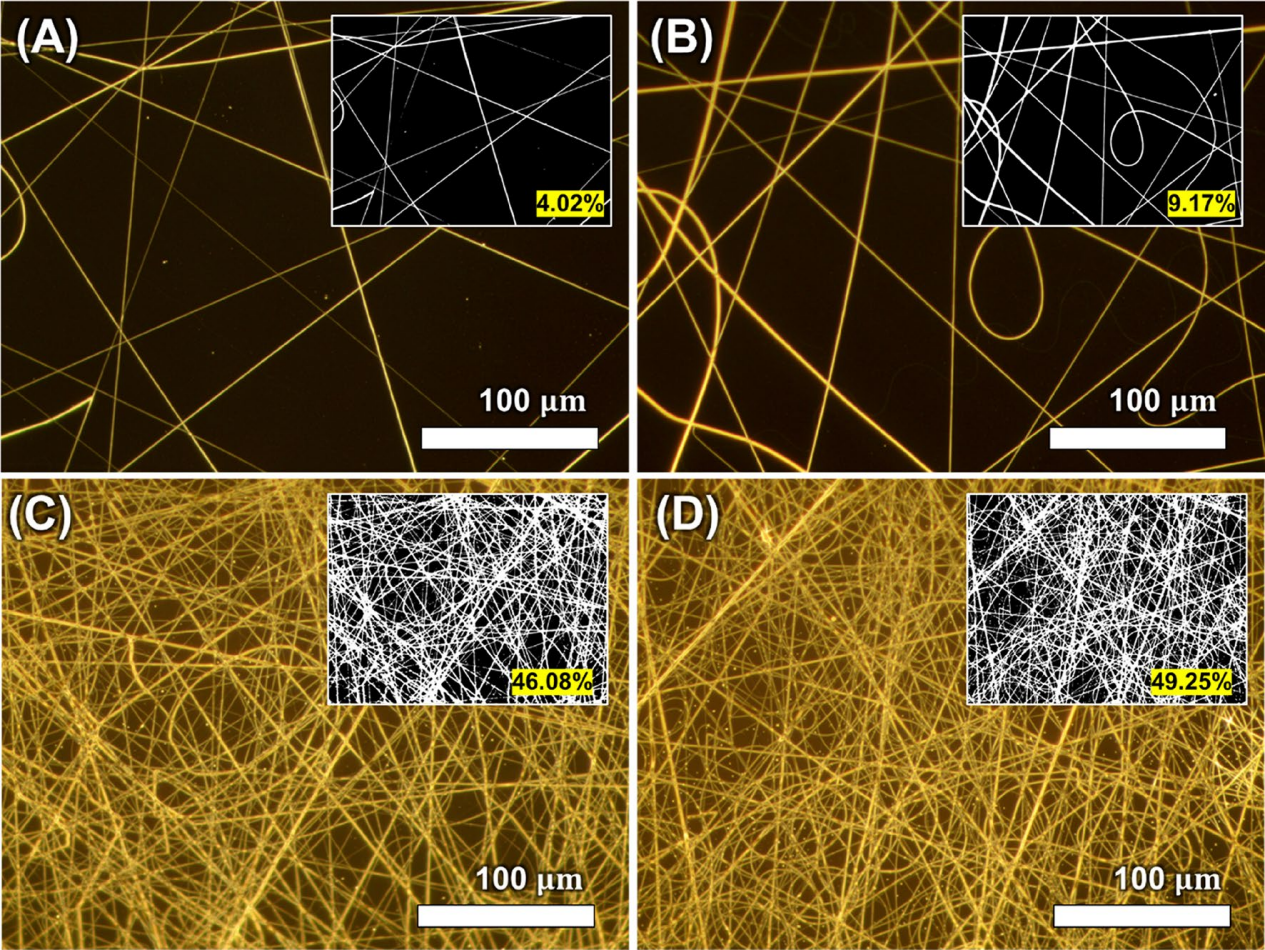
Comparison of fiber density against the electrospinning time: (**A**) 5 s, (**B**) 10 s, (**C**) 30 s, and (**D**) 60 s. The small numbers and images illustrate the mean ratio of the area occupied by the nanowire to the total area, and the black-and-white image (n = 5).

Electrospun nanofibers are subsequently metallized via an electroless deposition process and transformed into microheaters. These microheaters operate on the principle of Joule heating, where thermal energy is produced upon the application of external electrical energy^53^. High performance in microheaters can be evaluated by their ability to generate high temperatures under low power^54^. Dense geometry in the nanofibers is beneficial for thermal stability and heat transfer, as an increase in the percentage of the metal fiber network deposited on the substrate results in a higher generation of thermal energy^55^. Figure 3 presents an optical microscope image that shows the distribution of PVP-palladium nanofibers collected on a silicon nitride membrane as a function of electrospinning time. Since the quantity of fibers collected varied with the electrospinning time, we compared them to ascertain optimal conditions. The optical image was digitized with a threshold to compute the fiber distribution ratio. As shown in Fig. 3A–D, the ratio of the area occupied by the nanowire to the total area was 4.02%, 9.17%, 46.08%, 49.25% for electrospinning time of 5, 10, 30, 60 s, respectively. As demonstrated in Fig. 3C, a sufficient number of nanofibers were efficiently collected at 30 s.

**Figure 4:**
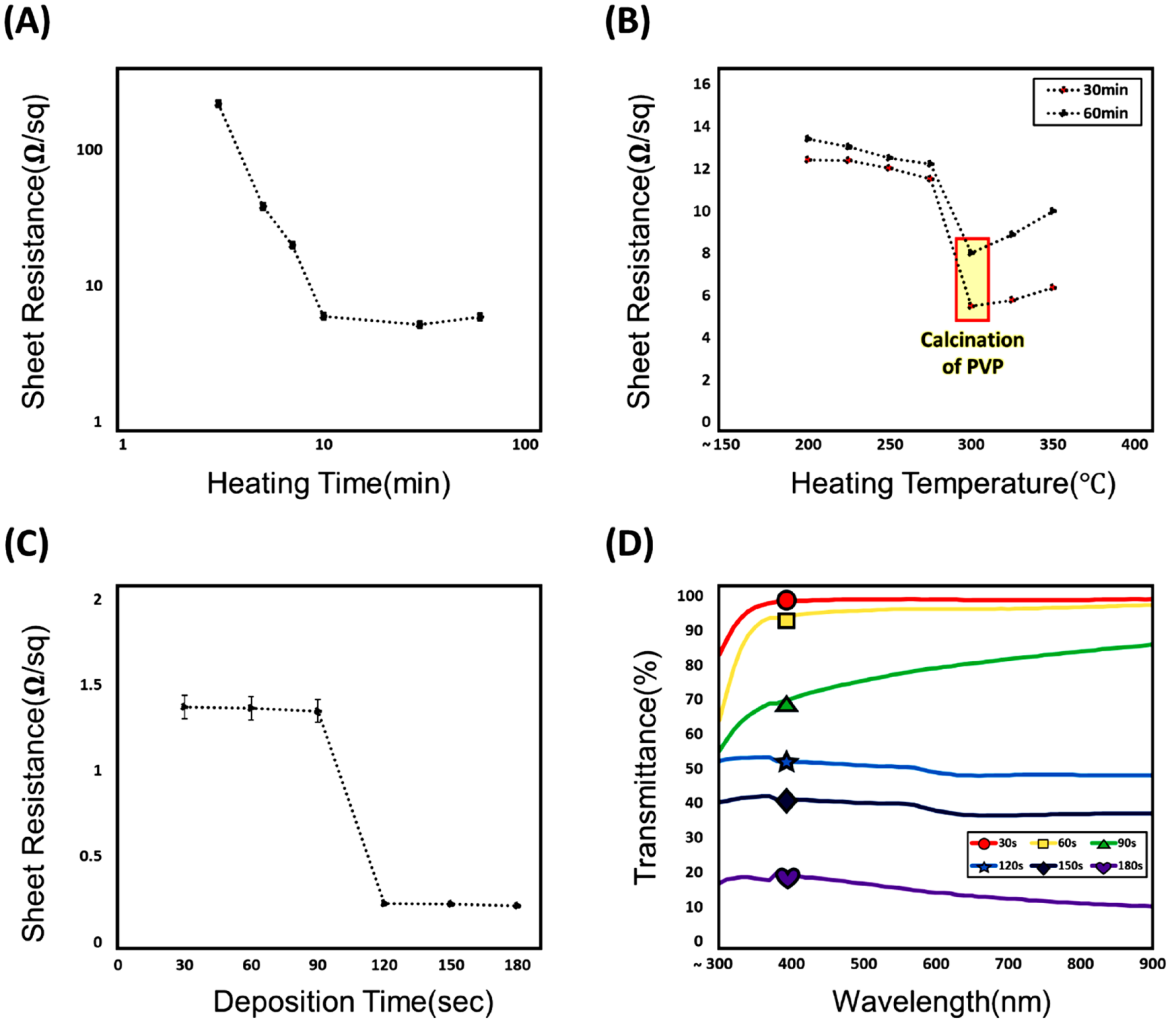
Analysis of the electrical and optical properties of the microheater. (**A**) Correlation between sheet resistance and heating time (log scale). (**B**) Correlation between sheet resistance and heating temperature. (**C**) Correlation between sheet resistance and transmittance in the visible range of 300–900 nm. (**D**) Correlation between sheet resistance and electroless deposition time. n = 5. Error bars represent the standard error.

Figure 4 presents the electrical and optical characteristics of fabricated microheaters. Figure 4A shows the variation in sheet resistance of the microheater with respect to heat treatment time on a logarithmic scale. From 3 to 10 min, the sheet resistance of the microheater decreased rapidly with increasing heat treatment time, eventually saturating at 5.71 Ω/sq, representing an 89.9% decrease. This trend was attributed to the elimination of intersections between nanofibers during heat treatment, and the formation of a seed layer with stably embedded palladium as the PVP component in the fibers decomposed due to heat.

Figure 4B shows the variation in sheet resistance of the microheater with respect to heat treatment temperature and time. The blue and red dots represent heat treatment times fixed at 30 and 60 min, respectively, with the heat treatment temperature rising from 200 to 350 °C. For 30 min at 200–300 °C, the microheater’s sheet resistance decreased from 12.42 to 5.5 Ω/sq as the annealing temperature increased, before increasing to 6.38 Ω/sq at 350 °C. Similarly, for 60 min at 200–300 °C, the microheater’s sheet resistance decreased from 13.39 to 8 Ω/sq as the annealing temperature increased, but then rose to 9.98 Ω/sq at 350 °C. This suggests that overlaps between nanofibers and PVP components are effectively removed and decomposed as the heat treatment temperature rises. The calcination of PVP starts from 300 °C so that the Pd ions embedded into the PVP fibers starts to actively react with copper ions in electroless deposition solution. However, when the heating time is too long or heating temperature is too high, the PVP fibers were completely calcinated^56^. Therefore, the Pd ions and copper ions reacts more actively and the amount of gas bubble containing hydrogen in the deposition solution becomes larger^57^. These can cause the low adhesion between the deposited copper film and the substrate, so that the sheet resistance of the nanofiber starts to decrease^58^.

Figure 4C shows the variation in sheet resistance of the microheater with respect to the copper electroless deposition time. From 30 to 90 s, the microheater’s sheet resistance gradually decreased from 1.36 to 1.34 Ω/sq with increased deposition time, and then sharply fell to 0.22 Ω/sq after 120 s. The sheet resistance at 150 and 180 s was 0.2212 Ω/sq and 0.2111 Ω/sq respectively. Compared to sheet resistance at 120 s, the deviation was small as well. And we confirmed that the sheet resistance started to saturate at 120 s of deposition time. The sharp decrease of sheet resistance depending on electroless plating time can be explained as follows. The copper deposition rate and thickness of copper in electroless deposition process can be adjusted by deposition time, amounts of additives, pH, temperature and so on. Nucleation of copper particle begins on the surface of the Pd catalytic site and as time increases, copper three dimensional crystallites (TDCs) enter bulk stage^59^. Therefore, the fully grown TDC makes the linewidth of the copper growing on the Pd embedded seed layer increase, leading enhanced electrical conductivity^60^.

Figure 4D presents the transmittance of the microheater as a function of copper electroless deposition time, across a wavelength range of 300–900 nm. When electrospun nanofibers of the same density were electrolessly plated for 30, 60, 90, 120, 150, or 180 s, the microheaters exhibited average transmittance of 98.09%, 94.44%, 77.2%, 49.8%, 38.61%, and 14.50%, respectively. As the electroless deposition time increased, the quantity of copper deposited increased, resulting in a gradual decrease in transmittance. From Fig. 4C,D, it is evident that the sheet resistance of the microheater and the transmittance in the visible region can be controlled by adjusting the electroless deposition time.

**Figure 5:**
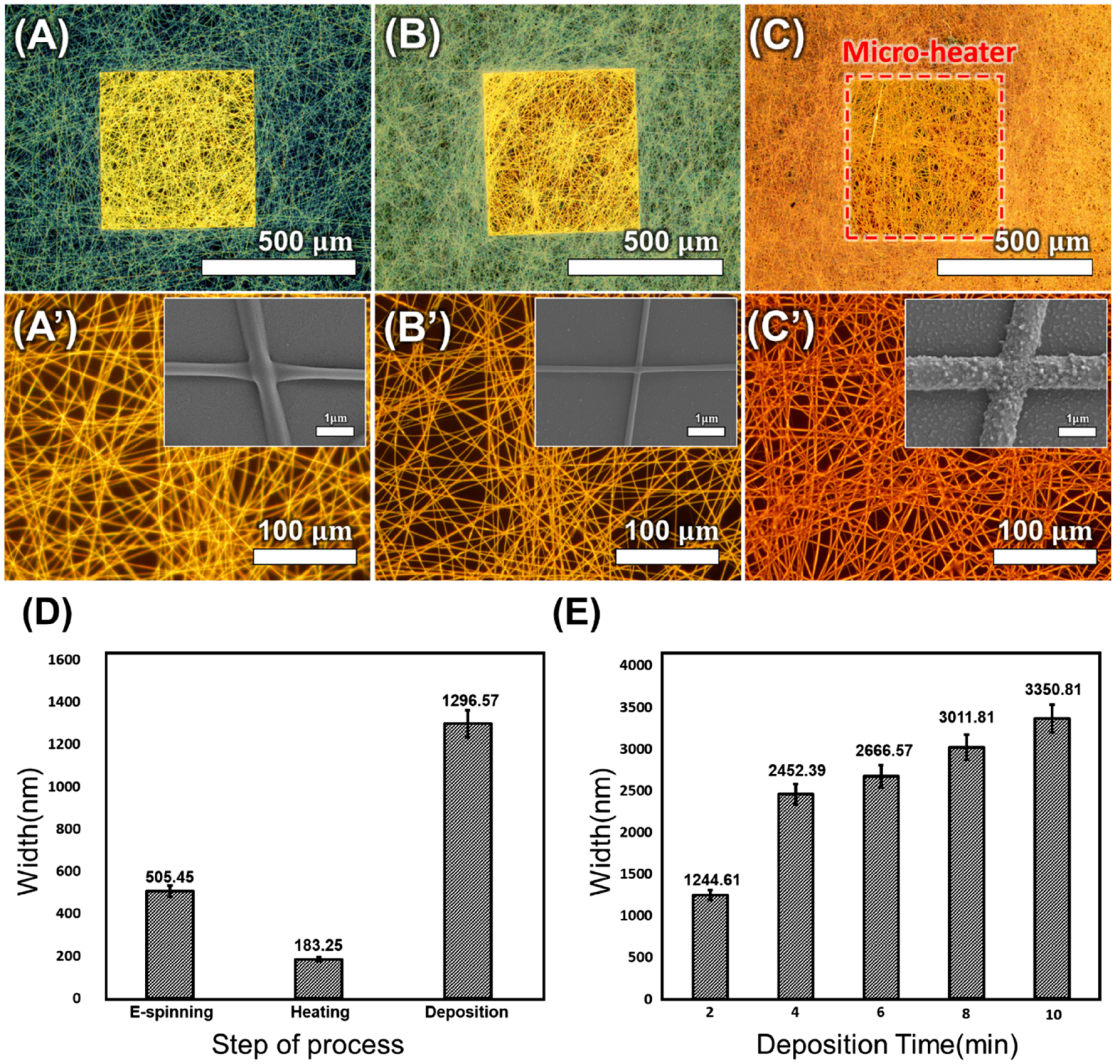
Analysis and morphologies of the nanofibers depending on the overall fabrication steps. (**A**,**A**′) Optical images of the electrospun PVP-palladium nanofibers. (**B**,**B**′) Optical images of the palladium-embedded seed layer heated at 300 °C for 30 min. (**C**,**C**′) Optical images of electroless Cu-deposited nanofibers. The small images embedded on Fig. (**A**′,**B**′,**C**′) are the FE-SEM images of each process, respectively. (**D**) Copper nanofiber width at each process step. (**E**) Correlation between electroless deposition time and copper nanofiber width. n = 5. Error bars represent the standard error.

Figure 5 presents the changes in nanofiber morphology during the fabrication stages of the microheater. Figure 5A,A′ shows the morphology of PVP-palladium nanofibers electrospun onto a silicon nitride membrane. Figure 5B,B′ presents the seed layer with a reduced linewidth due to the thermal degradation of PVP. The intersections occurring between nanofibers were also eliminated^61^. Figure 5C,C′ illustrates the nanofiber network fabricated through 2 min of electroless deposition, with a significant increase in linewidth. Figure 5D demonstrates the variation in nanofiber linewidth according to the microheater fabrication steps. The PVP-palladium nanofibers fabricated by electrospinning averaged 505.45 nm, which decreased to 183.25 nm after heat treatment due to PVP decomposition. After 2 min of copper electroless deposition, the linewidth expanded to 1296.57 nm. Figure 5E shows the variation in nanofiber linewidth with copper electroless deposition time.

### Performance evaluation of microheater

**Figure 6:**
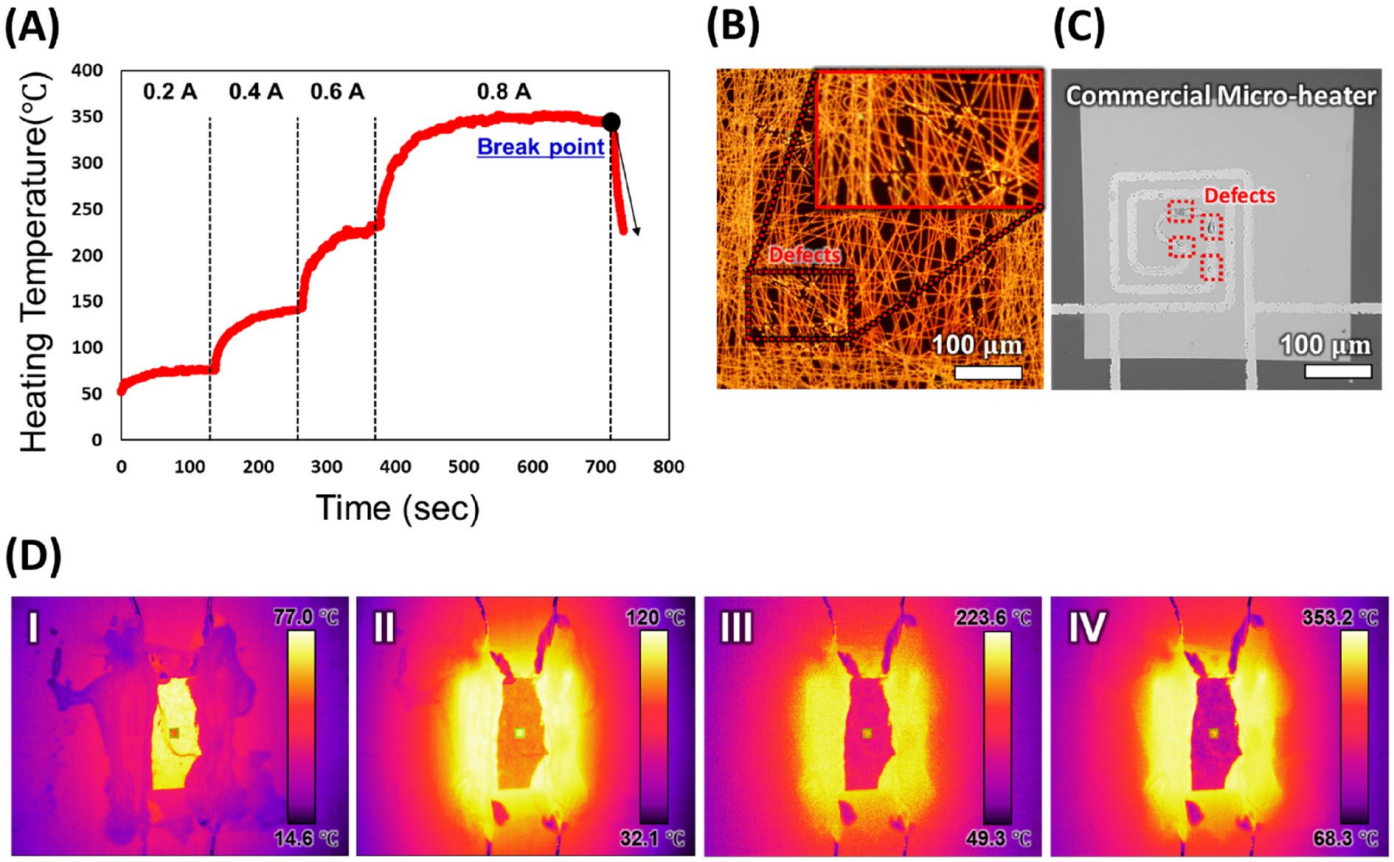
Performance evaluation of the microheater. (**A**) Correlation between the duration of applied current and microheater temperature. Various currents were applied to the microheater, and the resulting temperature changes were recorded. The dotted line represents the time of constant current change. (**B**) Optical microscope image of the microheater with defects. (**C**) Optical microscope image of the commercial microheater with defects. (**D**) Infrared images of the microheater at about (I) 75 °C, (II) 120 °C, (III) 220 °C, and (IV) 350 °C.

Figure 6 presents an evaluation of the final microheater performance. Figure 6A shows the temperature of the microheater as a function of current. When a constant current was applied, the temperature rose and then remained stable within less than 5 s; the average temperatures recorded were 75 °C, 120 °C, 220 °C, and 350 °C when the current of 0.2 A, 0.4 A, 0.6 A, 0.8 A was applied, respectively. We calculated the time interval from when the heater first reached at 350 °C and maintained, and the maintained time of heating was about 180 s. Then, the microheater reached a maximum temperature of 353.2 °C at a current of 0.8 A and broke up.

Figure 6B is an optical microscope image of microheater with defects. The copper nanowires were damaged by the mechanical stress, but their heating performance was not degraded. Because they are strongly connected with other wires around, so the electrical performance didn’t decrease even if a one disconnection of wire happens. Also, the copper network was oxidized by the heat, but they did not deform or broke, as same as before heated up (Supplementary Fig. 1). However, the localized heat caused the copper film to break, and the temperature starts to decrease. The failure of the microheater fabricated in this study is the result of a sharp temperature gradient between the window and frame parts of the silicon nitride membrane substrate. As shown in Fig. 6D, when current was applied in each step to the microheater, the temperature of microheater increased, while the temperature gradient between window and frame parts of it is large. Since the thickness of window part is 100 nm and the thickness of frame part is 200 μm, there is a difference in thermal conductivity, which leads to a sharp temperature gradient when current was applied. This locally concentrated heat gradually damages the thin window parts of microheater and eventually failure begins.

Figure 6C is the optical image of commercial heater with defects. We compared the microheater fabricated in this study and the commercial microheater fabricated based on MEMS process (Company named ‘A’). The window size, frame size and the thickness of commercial heater was as same as microheater of this study. The commercial heater has serpentine Au pattern in the silicon nitride substrate. When the applied current was 25 mA it was heated up to 220 °C and then broke (Supplementary Fig. 2). Compared with microheater fabricated in this study was heated up to 353 °C when 0.8 A of current was applied, these results suggest that the commercial heater is much less durable. Since the commercial microheater is consisted of single line metal conductor, when the defect happens due to the heat concentration, then the microheater immediately broke down and temperature decreases. But the microheater fabricated through electrospinning and electroless deposition didn’t broke easily even though defects happen to heater because the copper wires are connected strongly in a network form. In addition, for manufacturing commercial heaters, it needs precise control to align the conductive pattern on the substrate which leads to high manufacturing cost.

Figure 6D shows the final temperature distribution across each section during current application, captured using an infrared camera. In Fig. 6D I, II, III, and IV, maximum temperatures of 77 °C, 120 °C, 223.2 °C, and 350 °C were achieved in each section at a current of 0.2, 0.4, 0.6, and 0.8 A, respectively. The temperature was measured only for the window area of the microheater. When the current was applied to the microheater, the temperature increases from the frame area firstly, which is adjacent to the macro electrode used to connect to the microheater (Fig. 6D-I). And then the temperature of the window area increases rapidly as the applied current was gradually increased (Fig. 6D-I, II, IV). As shown in the infrared image, both window area and the frame area has uniform thermal distribution. This process enabled the fabrication of a high efficiency microheater with a fast thermal response and high heating temperature, even at low power.

Also, we evaluated the performance of the microheater fabricated in this study by calculating the figure of merit (FoM), which is one of the ways to evaluate the performance of transparent electrodes. The relationship between ${{\text{T}}}_{{\text{R}}}$ and ${{\text{R}}}_{{\text{S}}}$ is verified using figure of merit (FoM), definded by the relational equation, where FoM = ${\sigma }_{dc}/{\sigma }_{op}$(the ratio of electrical conductivity to optical conductivity)^62^.

$${{\text{T}}}_{{\text{R}}}={(1+\frac{188.5}{{R}_{S}\times FoM})}^{-2}$$

A high-performance transparent electrode has a high FoM when it has high ${{\text{T}}}_{{\text{R}}}$ and ${{\text{R}}}_{{\text{S}}}$ both. The FoM of the microheaters fabricated in this study ranged from about 2000–13,000, with the highest FoM being 13,860. These values are significantly higher compared to previously studied transparent electrodes. Because the copper nanofiber network fabricated based on electrospinning and electroless deposition makes the electrode having high optical transmittance and low electrical resistance rather than other metal patterning techniques.

## Conclusion

In this study, we fabricated a microheater with a nanoscale pattern on a thin silicon nitride membrane through three steps: electrospinning, heat treatment, and electroless deposition. Given that all three steps are carried out at atmospheric pressure, this method is simpler than conventional metal patterning techniques such as PVD and CVD. The process is also well-suited for mass production due to the large-area applicability of electrospinning. Notably, the nanofiber web generated by electrospinning undergoes metallization via an electroless deposition process, which is performed without the need for a separate power source. This offers a major advantage as it eliminates the need for separate masking, etching, and control technologies.

Additionally, the formation of a web structure by fibers with nanoscale diameters endows the microheater with superior electrical efficiency and high mechanical strength compared to commercial microheaters that feature a single conductor structure. We found that the electrical and optical properties of the microheater could be easily modulated by controlling factors such as the nanofiber density, electrospinning duration, heat treatment time and temperature, and electroless deposition duration. The final microheater exhibited rapid thermal response characteristics and high heating performance, with a maximum temperature of approximately 350 °C as determined by comparing temperature changes under various currents.

In the future, we anticipate that microheaters for various applications will be fabricated by modifying the geometry of the electrospun nanofiber web, possibly by introducing a method such as passivation masks, or enhancing the performance of the microheater through the application of different metals like gold, silver, or tungsten. The microheaters and fabrication process discussed in this study hold promise for use in applications requiring localized heating such as gas sensors, electron microscopes, fuel cell heat sources, and electronics heating. We expect that these applications could further extend to advanced areas such as solar cells, displays, and wearable devices when deployed on flexible transparent substrate materials.

### Supplementary Information

Supplementary Figures.

## Data Availability

The datasets generated during the current study are available from the corresponding author on reasonable request.
