# Supplementary material for: Microheater with copper nanofiber network via electrospinning and electroless deposition
Source: Sci Rep. 2023 Dec 14;13:22248. doi: 10.1038/s41598-023-49741-7 (PMC10721892; doi:10.1038/s41598-023-49741-7)
Supplement: Supplementary file 1 — Supplementary Figures. [file 41598_2023_49741_MOESM1_ESM.docx]

**Microheater with Copper Nanofiber Network via Electrospinning and Electroless Deposition**

Na Kyoung Kim^1^, Kanghyun Kim^2^, Hansol Jang^3^, Hyun-Joon Shin^3^, Taechang An^4^,

Geon Hwee Kim^1,#^

^1^Department of Mechanical Engineering, Chungbuk National University (CBNU), 1, Chungdae-ro, Seowon-gu, Cheongju-si, Chungcheongbuk-do, 28644, Republic of Korea

^2^Department of Mechanical Engineering, Pohang University of Science and Technology (POSTECH), 77, Cheongam-ro, Nam-gu, Pohang-si, Gyeongsangbuk-do, 37673, Republic of Korea

^3^Department of Physics, Chungbuk National University (CBNU),1, Chungdae-ro, Seowon-gu, Cheongju-si, Chungcheongbuk-do, 28644, Republic of Korea

^4^Department of Mechanical & Robotics Engineering, Andong National University (ANU), 1375, Gyeong-dong-ro, Andong-si, Gyeongsangbuk-do, 36729, Republic of Korea

^#^Corresponding author: geonhwee.kim@chungbuk.ac.kr (Geon Hwee Kim)


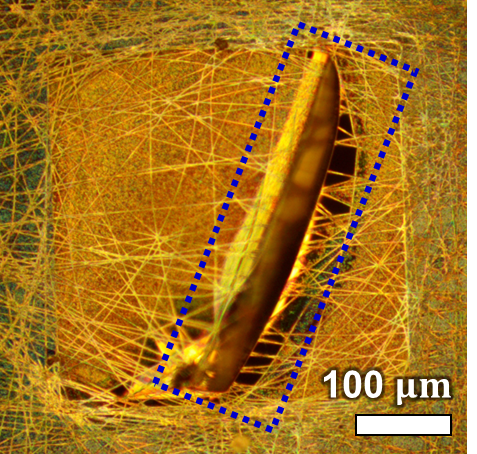
Supplementary Figure 1. Optical microscope image of the microheater fabricated in this study after broken.

**
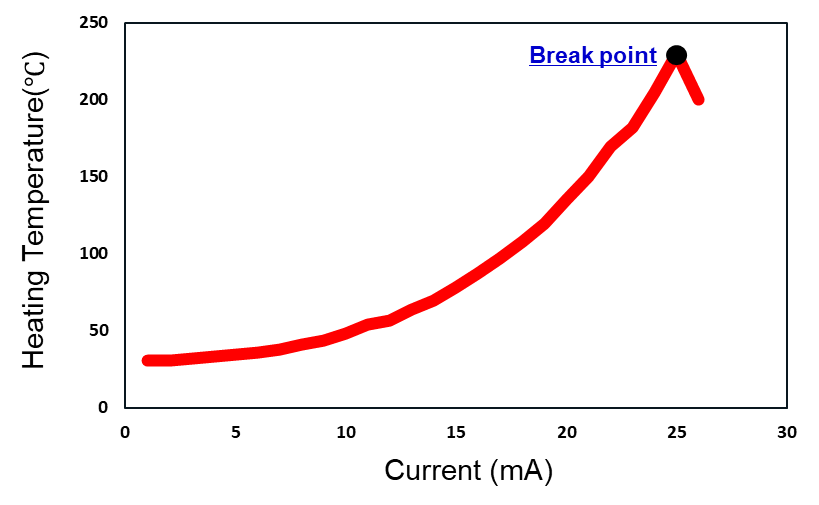
**

Supplementary Figure 2. Correlation between the applied current and temperature for commercial microheater (Company named ‘A’). The current was applied continuously, and the resulting temperature changes are recorded.
